# Computer simulation of optimal lipped polyethylene liner orientation against prosthetic impingement

**DOI:** 10.1186/s13018-022-03093-6

**Published:** 2022-04-04

**Authors:** Yi Hu, Xianhao Zhou, Hua Qiao, Zhenan Zhu, Huiwu Li, Jingwei Zhang

**Affiliations:** Department of Orthopaedics, Ninth People’s Hospital, Shanghai Jiao Tong University School of Medicine, 639# Zhizaoju Road, Shanghai, 200011 People’s Republic of China

**Keywords:** Range of motion, Elevated liner, Impingement, Total hip arthroplasty, Orientation, Computer simulation

## Abstract

**Background:**

Lipped or elevated acetabular liners are to improve posterior stability and are widely used in hip arthroplasty. However, concerns of increasing impingement exist when using such liners and optimal orientation of the elevated rim remains unknown. We aimed to identify the impact of lipped liner on the range of motion (ROM) before impingement and propose its optimal orientation.

**Methods:**

An isochoric three-dimensional model of a general hip-replacement prosthesis was generated, and flex-extension, add-abduction and axial rotation were simulated on a computer. The maximum ROM of the hip was measured before the neck impinged on the liner. Different combinations of acetabular anteversion angles ranging from 5 to 30 degrees, and lipped liner orientations from posterior to anterior were tested.

**Results:**

When acetabular anteversion was 10 or 15 degrees, placing the lip of the liner in the posterosuperior of the acetabulum allowed satisfactory ROM in all directions. When acetabular anteversion was 20 degrees, extension and external rotation were restricted. Adjusting the lip to the superior restored satisfactory ROM. When acetabular anteversion was 25 degrees, only placing the lip into the anterosuperior could increase extension and external rotation to maintain satisfactory ROM.

**Conclusions:**

This study showed that optimal lipped liner orientation should depend on acetabular anteversion. When acetabular anteversion was smaller than 20 degrees, placing lip in the posterior allowed an optimally ROM. When acetabular anteversion was greater than 20 degrees, adjusting lip to the anterior allowed a comprehensive larger ROM to avoid early impingement.

**Supplementary Information:**

The online version contains supplementary material available at 10.1186/s13018-022-03093-6.

## Introduction

Dislocation is the first reason for revision total hip arthroplasty (THA) and recurrent dislocation occurs in 60% of patients after a first dislocation and more than half of them need re-revision [1, 2], which seriously deteriorates joint function and increases social financial burdens [3, 4]. Therefore, surgeons must be aware and deal well with factors which may bring postoperative joint instability, including patient general condition, surgical technique and implant characters, to avoid dislocation as much as possible after primary THA [5].

Impingement is the contact between metal femoral neck and cup liner or bone-to-bone contact and is related to polyethylene wear and subsequent dislocation [6]. Accurate acetabulum position is integral to avoid these complications and is greatly studied [7–9]. However, previous studies often ignored polyethylene liner which was also an important part of the whole acetabular system. Lipped liner has an elevated rim of 10–20 degrees, which is designed to be placed in the posterosuperior acetabulum to decrease posterior dislocation rate by restricting femoral head movement through a hemisphere over 180 degrees and is widely used in primary THAs [10, 11]. However, although it is generally accepted that lipped liner should be placed posterosuperiorly, it has not been proven insufficiently [12, 13]. Besides, concerns of component impingement, wear and future loosing exist when using such liners with over 81% of retrieved lipped liners showing impingement signs [10, 14, 15].

As the inability to identify liner radiographically, computer simulation is used as a protocol to trace liner impingement [16]. Since impingement causes joint instability and continuing movement leads to dislocation, range of motion (ROM) before impingement reflects safe motion zone. Although dislocation risk is expected to be reduced with lipped liner increasing femoral head jumping distance, it happens after impingement and acquiring a wider ROM before impingement is important to decrease possible polyethylene wear. Thus, we intended to use computer simulation to investigate the influence of different combinations of acetabulum anteversion degrees and lipped liner orientations on ROM before impingement. We tried to answer: (1) Would the ROM before impingement meet the requirements of daily activities when a lipped liner was used? (2) When acetabulum anteversion was changed, where should lipped liner be placed to obtain a maximum ROM before impingement?

## Materials and methods

**Fig. 1:**
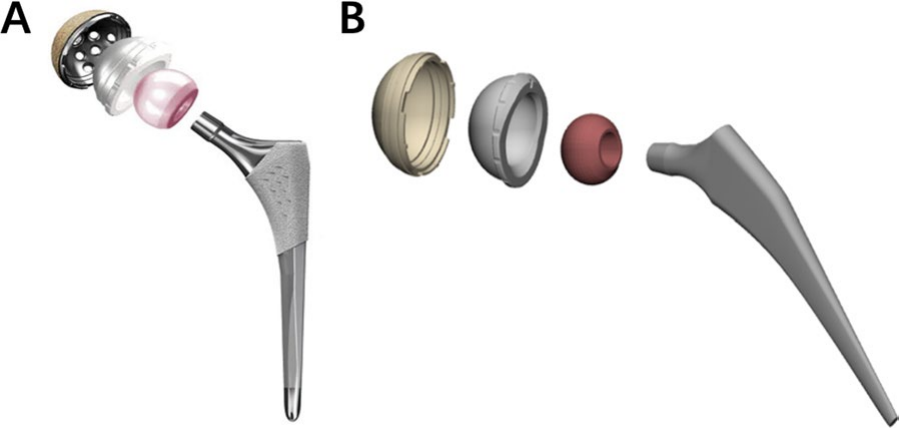
**a** The hip prothesis system to be modeled. The stem was Secur-Fit and the head was Biolox. The cup was Trident PSL and the liner was Trident X3 with a 10 degrees polyethylene elevated rim. **b** The isochoric three-dimensional model based on the prothesis

Our institutional review board approved this study. The isochoric three-dimensional model of a general hip-replacement prosthesis (Stryker Orthopaedics, Kalamazoo, Michigan, USA) was initially generated with the use of Rhino (Rhinoceros 6.0, Robert McNeel & Associates, USA) via sweeping and lofting (Fig. 1). The geometry parameters of the prosthesis used are listed in Table 1. A 10-degree lipped polyethylene liner was used. The model was of the same size and geometry characters with the hip prosthesis system.

**Table 1:** Geometry characters of the prothesis

Geometry character	Length or angle (mm/°)
Acetabular diameter	50
Head size	28
Neck length	30
Head-to-neck ratio*	1.54–1.72
Neck-to-shaft angle	132°
Stem length	140

*The cross section of the neck was an ellipse which was shown in supplementary material (see Additional file 1). We used the long axis and the short axis to calculate the head-to-neck ratio separately

**Fig. 2:**
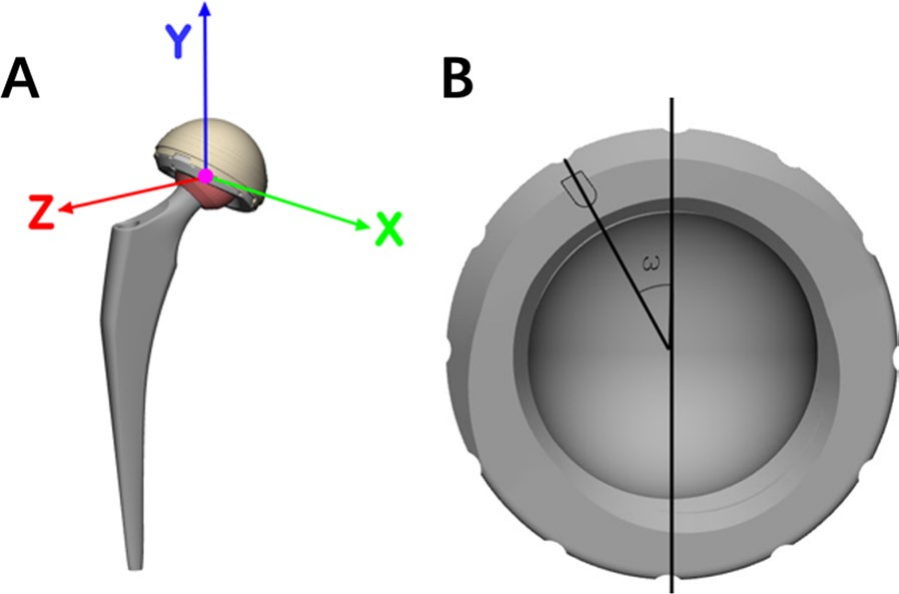
**a** The coordinate system determined by the International Society of Biomechanics. **b** The view of the acetabular opening plane to show the orientation of the elevated rim. One line connected the center of the acetabular circle and the midpoint of the elevated rim. The other line was intersected by the acetabular opening plane and the y–z axial plane. The angle ω formed by these two lines was the orientation of the elevated rim

The three-dimensional model was imported into Materialise 3-matic Medical 11.0 (Materialise, Leuven, Belgium) to mimic different motions. The coordinate system and the axes around which motion was described were determined according to the recommendations made by the International Society of Biomechanics [17]. The origin of hip axes was located at the center of the head and it was also the center of the acetabulum. With the hip in the neutral position, the x-axis pointed anteriorly and was perpendicular to the vertical axis of the stem. The y-axis pointed superiorly and was parallel to the vertical axis of the stem. The z-axis pointed to the patient’s right side (Fig. 2). Flex-extension of the hip was described around the z-axis, with axial rotation around the y-axis and add-abduction around the x-axis. Acetabular anatomical anteversion and abduction was adopted [18]. The femoral anteversion was measured as the angle between the femoral neck axis and the y-z axial plane. The orientation of the elevated rim was determined by the angle formed by the midline of the elevated rim and the line intersected by the acetabular opening plane and the y–z axial plane (Fig. 2). Negative degrees represented the lipped liner pointing posteriorly, and positive degrees represented the lipped liner pointing anteriorly. We used − 30, 0 and 30 degrees to represent lipped liner pointing to the posterosuperior, superior and anterosuperior, respectively.

**Fig. 3:**
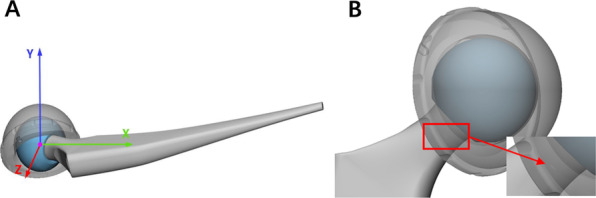
**a** Flexion was simulated and was observed from side. **b** Impingement on the liner of the cup during flexion was detected visually from a horizontal view and the flexion angle was recorded once impingement being visible. The red block showed the impingement area and the red arrow pointed to the contact between the femoral neck and the cup liner

The ROM was measured by moving the femur in the desired direction until the neck visually impinged on the liner of the cup (Fig. 3). This method was consisted with the study before and proved to have enough accuracy [19]. The computer software was capable of detecting this degree, and the maximum ROM before impingement was defined as the number of it. The acetabular anteversion angles of 5, 10, 15, 20, 25 and 30 degrees were studied in combination with the lipped liner pointing at − 30, 0, and 30 degrees. Flexion, extension, add-abduction, axial rotation and internal rotation at hip 90 degrees flexion were measured for all above combinations. The cup abduction was fixed to 50 degrees for an approximate 40 degrees total acetabulum abduction when using a 10-degree lipped liner and the stem anteversion was 15 degrees without changing during the whole test.

To determine the effect of different combinations of prosthetic positions on ROM, each ROM was classified according to two zones: excellent and poor. An excellent ROM allows greater than 110 degrees of flexion, 30 degrees of extension, 30 degrees of adduction–abduction, 30 degrees of internal-external rotation and 30 degrees of internal rotation at 90 degrees hip flexion. A poor ROM means less than 110 degrees of flexion, 30 degrees of extension, 30 degrees of adduction–abduction, 30 degrees of internal-external rotation and 30 degrees of internal rotation at 90 degrees hip flexion. The excellent zone met the requirements of daily activities [20]. We used Prism (GraphPad Prism 8 for Mac, GraphPad Software, San Diego, CA, USA) to generate graphs.

## Results

**Fig. 4:**
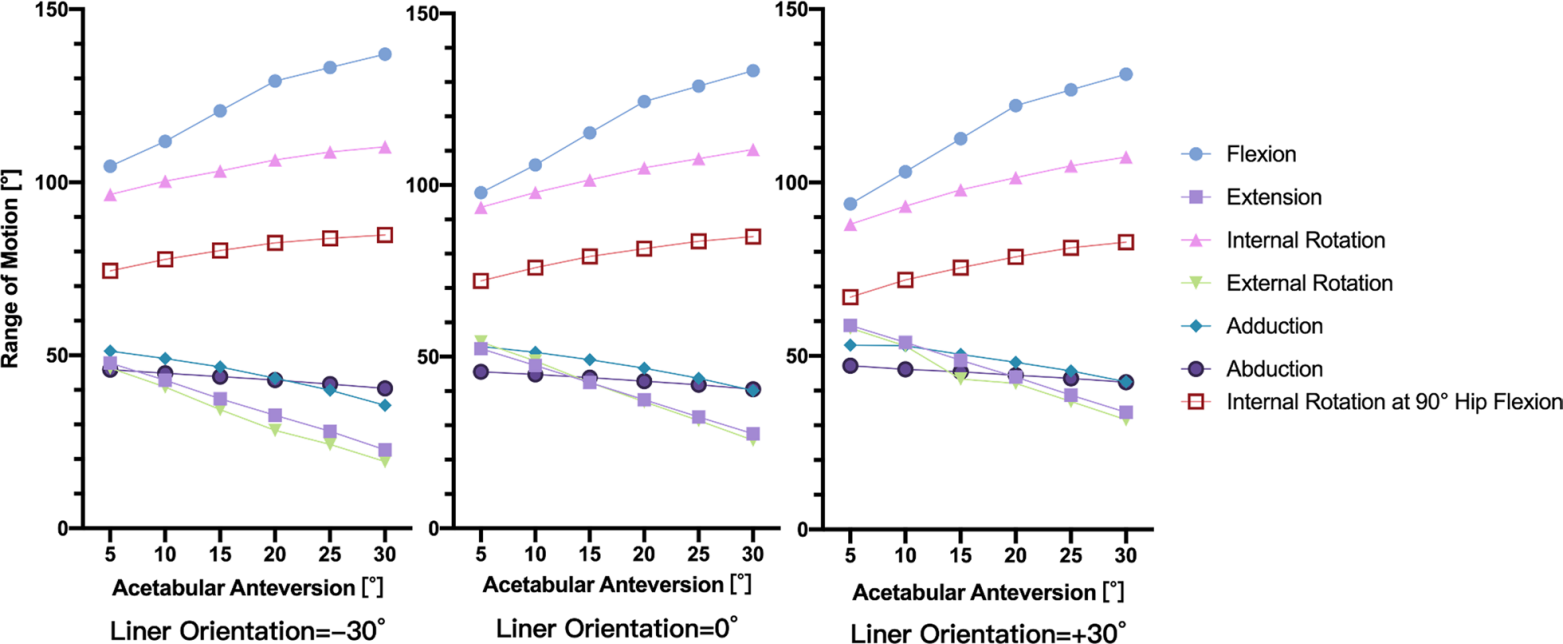
Range of motion under different combinations of acetabular anteversion degrees and lipped liner orientations

Overall, increasing acetabular anteversion angles increased hip flexion, internal rotation, internal rotation at hip 90 degrees flexion and decreased extension, external rotation and adduction–abduction. Placing the lip of the liner in the anterosuperior increased extension, external rotation, adduction and decreased hip flexion, internal rotation. Placing the lip anterosuperiorly did not have much influence on abduction (Fig. 4).

When the lip of the liner was placed in the posterosuperior quadrant of the acetabular component and the acetabular anteversion was 10 or 15 degrees, it allowed hip flexion over 110 degrees, extension over 30 degrees and internal rotation over 80 degrees at hip 90 degrees flexion. This ROM met the demand of daily activities and the use of a lipped liner did not restrict hip movements.

At 10 or 15 degrees of acetabular anteversion, placing the lip of the liner in the posterosuperior brought an excellent ROM. If the lip was placed in the superior or anterosuperior, although extension and external rotation was increased, hip flexion was decreased to less than 110 degrees, which made the ROM poor.

However, at 20 degrees of acetabular anteversion, if the lip was still placed in the posterosuperior, extension and external rotation would be restricted to less than 30 degrees. If the lip was adjusted to the superior quadrant of the acetabulum in the above situation, although hip flexion was slightly decreased, it still reached over 110 degrees and extension and external rotation was restored to over 30 degrees, which made the ROM excellent again.

When the acetabular anteversion was 25 degrees or even larger, posterosuperiorly placed lip even decreased extension and external rotation to less than 20 degrees. When the lip was placed in the superior, extension and external rotation reached slightly over 30 degrees. Only when the lip was placed in the anterosuperior, extension and external rotation could be restored to over 30 degrees and flexion still reached over 110 degrees. It turned the ROM excellent again.

In above situations, hip adduction–abduction was over 40 degrees and internal rotation was more than 80 degrees. Internal rotation at hip 90 degrees flexion was all over 70 degrees. These three motions met the requirements of daily activities and were not included into analysis.

## Discussion

Our study was designed to characterize impingement patterns associated with various components’ locations and orientations. Prosthetic impingement determines the functional end point of the stable ROM after a hip arthroplasty and is associated with wear, loosing and long-term failure [6]. Therefore, optimum positioning of the components is necessary to avoid a decrease in the stable ROM due to prosthetic impingement.

Lipped liner, as an important part of the acetabular system, is widely used to decrease the tendency of dislocation. However, there are concerns of elevated rim polyethylene liner increasing rate of impingement and there are few studies about it [11]. Our study initially put elevated rim polyethylene liner into consideration and found that lipped liner placed in the posterosuperior quadrant of the acetabular component did not decrease the ROM before impingent when the acetabular anteversion was 10 or 15 degrees. When the acetabular anteversion was 15 degrees, posterosuperiorly placed lipped liner allowed over 110 degrees of flexion and over 30 degrees of extension, which met the demand of daily activities [20]. Our result probably explained some clinical outcomes that posterosuperiorly placed lipped liner did not increase the cumulative probability of revision because of loosening compared to standard liner [15, 21].

The elevated rim of the liner should be placed in the posterosuperior conventionally. Lipped liners were designed by Charnley in the early 1970s to decrease the tendency for posterior dislocation of the femoral head [22]. The lipped lip increases stability by increasing the displacement required for the prosthetic head to dislocate [21], and it becomes apparent to place the liner in the posterior quadrant when using a posterolateral approach. Using a 15-degree liner in the posterior quadrant increases the internal rotation range of movement by 8.9 degrees without causing dislocation [23]. Our result suggested that when the acetabular component was in 10 to 15 degrees of anteversion, placing the lipped liner in the posterosuperior achieved enough ROM in all directions without increasing impingement risk compared to lipped liner placed in other directions. A relatively small acetabular anteversion made posterior dislocation easy [24] and our result supported the theory of placing the liner in the posterosuperior to increase stability by achieving the largest ROM before impingement. It proved the feasibility of placing the elevated rim in the posterosuperior quadrant of the acetabulum under common situation.

But our result also suggested that we should not place the lipped liner in the posterosuperior in all situations without changing. The optimum position of the elevated rim was depended on the orientation of the acetabular component. When the acetabular anteversion was more than 20 degrees, the ROM before impingement would be restricted by extension and external rotation less than 30 degrees which might cause anterior dislocation if the lipped liner was still placed in the posterosuperior. When the acetabular anteversion was more than 25 degrees, it became worse by extension and external rotation less than 25 degrees. However, if the lipped liner was transferred to the superior or anterosuperior in above situations, extension and external rotation would be restored to over 30 degrees while it had little effect on ROM on other directions. It suggested that when the acetabulum was placed in larger anteversion than common, an anteriorly or superiorly placed lipped liner might bring lager ROM before impingement. It has been reported that impingement was more frequently associated with an elevated anti-dislocation rim [25] liner and the predictive risk for dislocation was 6.6 times larger compared to standard liners when using an elevated rim liner for primary THA [26]. This might be that in these retrieved cases, the position of the elevated rim was not adjusted with the orientation of the acetabular component and was placed in the posterosuperior routinely. Yamaguchi et al. found an association between excessive cup anteversion combined with posteriorly placed elevated rim and impingement in retrieved liners [27]. Krushell et al. tested prosthetic ROM with acetabular component in different alignments and found elevated rim liners in satisfactorily positioned acetabular components offered no demonstrable benefit and the primary indication for these implants appeared to be in cases of instability due to acetabular malposition [28]. It was consistent with our study that the optimum position of the elevated rim depended on the orientation of the acetabular component.

Component-on-component impingement, followed by levering out of the femoral head, is by far the most common mode of dislocation in total hip arthroplasty [29]. Although ROM before dislocation does not equal to that before impingement [30], impingement should be minimized to reduce possible wear and loosing. Moreover, multiple confounding factors and sources of variability made it difficult to identify specific parameters influencing ROM before impingement. For example, implant position changes such as stem anteversion, neck-shaft angle and prosthetic head size have been proved to have an impact on no-impingement ROM [31] and it becomes more complex when taking in vivo factors into consideration. Our study first controlled other variables and explored the influence of the elevated rim of liner on ROM before impingement. The influence of the elevated rim of liner on dislocation deserves to be further studied.

There are a number of limitations associated with this study. First, this study focused on prosthetic impingement, while bony impingement also matters and there are other factors having an impact on prosthetic impingement including head size and head/neck ratio. However, given that there are so many confounding factors and bony anatomy characters vary, it is too complex to take all variables into consideration. We emphasized on the impact of the lipped liner on ROM by controlling other factors the same, and we offered a new insight into the optimum position of the elevated rim of the liner. Second, although this study was simulated on computer and might be different from in vivo conditions when taking soft tissue into consideration, it gave a general suggestion which might be still useful in vivo. Third, this study used only one kind of prosthesis. However, the prosthesis used in this study is of classical design and was applied widely. We believed that although values might be slightly changed in other kinds of prosthesis, the tendency of movement and the conclusion would be same.

## Conclusion

An optimally placed lipped polyethylene liner should depend on acetabular anteversion in total hip arthroplasty. When the acetabular anteversion is relatively small for about less than 20 degrees, placing the liner in the posterior quadrant of the acetabulum is acceptable. When the acetabular anteversion is more than 20 degrees, it is better to place the liner in the superior or anterior to achieve a comprehensive ROM to avoid early impingement.

## Supplementary Information

**Additional file 1**. The cross section of the neck was not a standard circle but an ellipse.

## Data Availability

The datasets used or analyzed during the current study are available from the corresponding author on reasonable request.

## Abbreviations

ROM: Range of motion
THA: Total hip arthroplasty
