# Supplementary figures and images for: Computer simulation of optimal lipped polyethylene liner orientation against prosthetic impingement
Source: J Orthop Surg Res. 2022 Apr 4;17:204. doi: 10.1186/s13018-022-03093-6 (PMC8981617; doi:10.1186/s13018-022-03093-6)

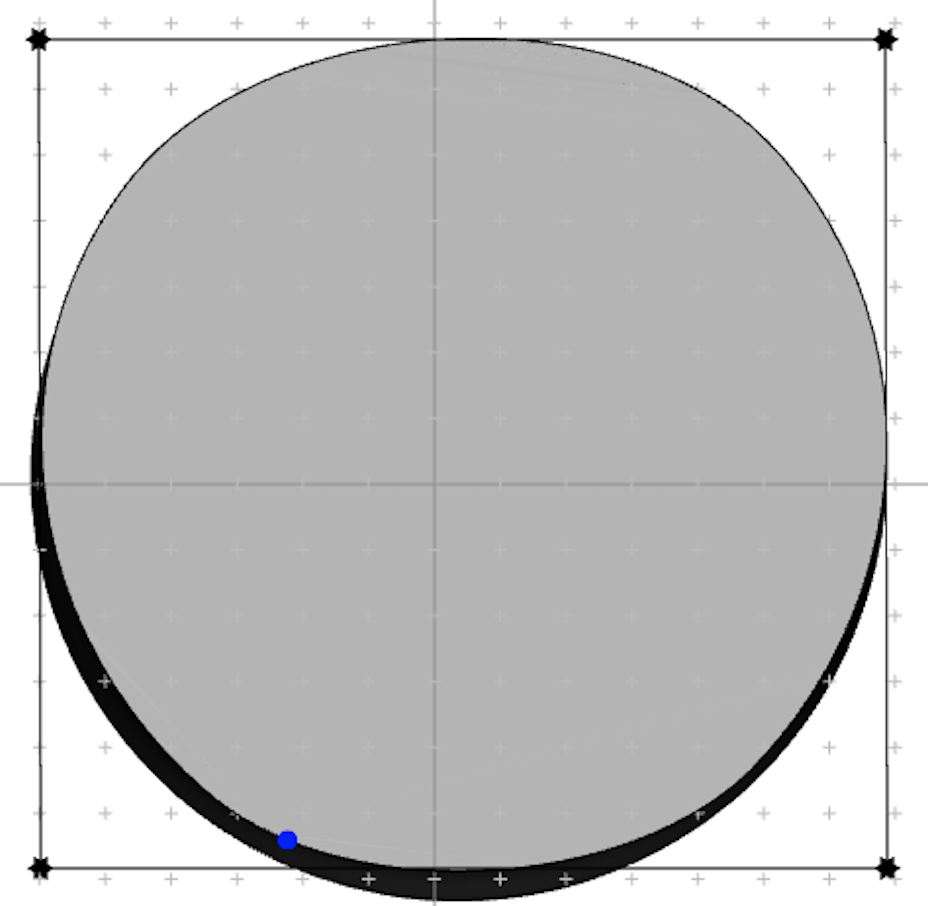

Supplement: Supplementary file 1 — Additional file 1. The cross section of the neck was not a standard circle but an ellipse. [file 13018_2022_3093_MOESM1_ESM.tiff]
